# Empyema Following Lobar Pneumonia

**Published:** 1900-11-17

**Authors:** 


### EMPYEMA FOLLOWING LOBAR PNEUMONIA.

It not infrequently happens that pneumonia is followed by empyema, and Dr. Hale White[1] says that by far the most important aid in diagnosing the existence of this sequence of events is the course of the temperature. The usual thing, if empyema follow, is for the temperature to fall when the crisis takes place, for it to remain down for two or three days, for it then to rise again, so that it soon becomes from 2° F. to 4° or 5° F. above normal in the evening and about 1° or 2° F. in the morning. This continues until the pus is evacuated.

The temperature, however, is not a sure guide in every case, for crisis only occurs in about half of all cases of pneumonia, and when there is no crisis the temperature sometimes falls irregularly and tediously. This irregularity, however, does not often show the considerable daily range which is so suggestive of pus. Other complications also may keep up the temperature. It must be remembered that an empyema following pneumonia may be quite a small one, and may be very difficult to find, requiring the insertion of the needle many times for its proper localisation.

It has often been stated that when the pus of an empyema contains a pure cultivation of pneumococci aspiration will suffice and incision is unnecessary. But the experience at Guy's is that, although after aspiration the temperature falls, it begins to rise again after a few days and assumes a hectic type, and the fluid re-accumulates, so that it would seem best, even where pneumococci alone are found, to incise, although cases may occur now and again in which a simple aspiration or perhaps a bursting through the lung may lead to recovery.

[1] Lancet, Nov. 10.

